# Efficacy of 5% topical minoxidil versus 5 mg oral biotin versus topical minoxidil and oral biotin on hair growth in men: randomized, crossover, clinical trial^☆^

**DOI:** 10.1016/j.abd.2023.07.008

**Published:** 2024-04-30

**Authors:** Flávia de Oliveira Valentim, Anna Carolina Miola, Hélio Amante Miot, Juliano Vilaverde Schmitt

**Affiliations:** Department of Infectology, Dermatology, Imaging Diagnosis and Radiotherapy, Faculty of Medicine, Universidade Estadual Paulista, Botucatu, SP, Brazil

Dear Editor,

The impact on the quality of life of those who suffer from hair disorders is comparable to those who have skin diseases such as psoriasis in plaques.^1^ On the other hand, individuals without any capillary disorder look for treatments to increase hair growth and thickness, in addition to strengthening the shaft from products marketed for this purpose but without any scientific proof.

Minoxidil is an established medication in the treatment of some hair disorders^2^, ^3^ and biotin is a vitamin that may interfere with the hair cycle.^4^, ^5^ However, there is a lack of literature data that supports biotin use with or without minoxidil to accelerate hair growth, especially in individuals without hair diseases.

We performed a study with the aim of evaluating the effectiveness of oral biotin 5 mg daily, application of topical 5% minoxidil twice daily, and associated use of both, during a period of 14 days each course, in increasing the speed of hair growth (HG).

A randomized, open, self-controlled, crossover clinical trial was performed. Ten healthy male participants, without hair disorders and not using any systemic or topical medication were recruited by convenience.

All participants were submitted to the three interventions (topical minoxidil only, oral biotin only, and both drugs combined). At inclusion, participants were allocated to all interventions, but in randomized sequence (crossover) performed by a computer program. Before starting medication use, a scalp shaving in the occipital region of a 1 cm^2^ area was made, followed by local phototrichoscopy, which was repeated after a mean time of 38 hours, in order to determine the baseline hg rate (HGR). The interventions were then applied for a period of 14 days. Immediately after, shaving and rephotographing the area 38 hours later was repeated to measure HGR under intervention. At the end of each cycle, the participants were reallocated between the remaining intervention groups. Therefore, all ten individuals participated in the three proposed interventions in a crossover protocol (Fig. 1). There was a 14 days washout interval between interventions.

**Figure 1:**
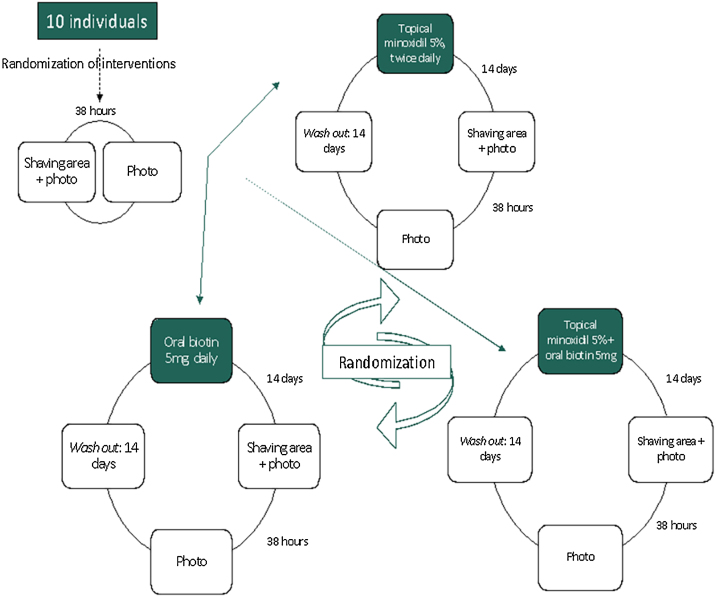
Flowchart of the proposed interventions, as well as the sequence of steps performed during the study, with the randomization of the order of interventions being performed prior to the beginning of the activities for each participant.

The occipital area was chosen by having a lower aesthetic impact on participants during the study and is less frequently affected by hair disorders, as we aimed to study healthy individuals’ hair. As the elimination half-life of minoxidil is four hours and biotin is two hours, the washout time of 14 days between interventions was regarded as sufficient to prevent the influence of previous medications on the subsequent results.

Each image obtained was analyzed by the ImageJ® software: ten anagen hairs’ length was measured in pixels, as well as the image area covered by hairs was evaluated using a software plugin to determine the HGR. 120 images were obtained and 1200 anagen hairs were analyzed. The average HGR was based on the difference between the lengths divided by time, in seconds, between the photographs. The difference in the percentual area of pictures covered by hair shafts was also divided by time to get the coverage area growth rate (Figure 2, Figure 3).

**Figure 2:**
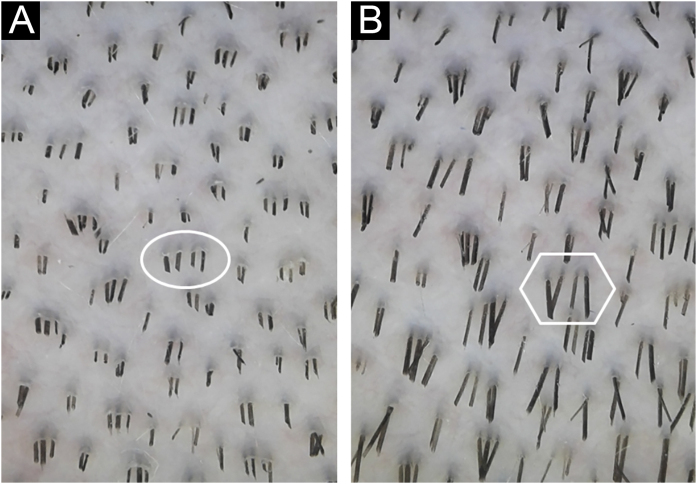
The same hair shafts’ length analyzed before (A) and after (B) 36 hours to obtain hair growth speed. * Ten individual shafts were measured in each picture, for each intervention.

**Figure 3:**
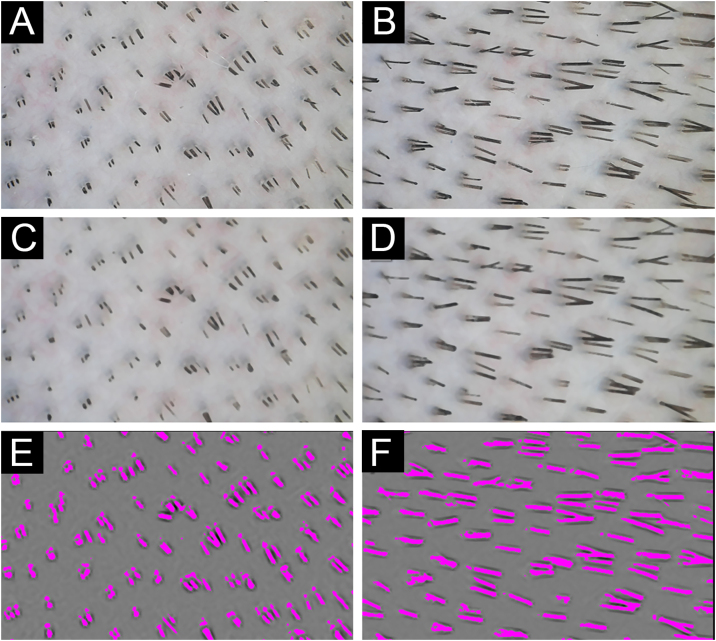
The hairs covering scalp area analyzed before (A) and after (D) 36-hours to obtain hair growth speed. * (A) Picture obtained before initially. (B) Picture A with median filtering. (C) Segmentation of the hairs from the initial picture in A. (D) Picture obtained 38-hours after A of the same area. (E) Picture D with median filtering. (F) Segmentation of the hairs from the initial picture in D.

Continuous variables were expressed as mean and standard deviation or median and quartiles after normality tests. The HGR and the area covered by shafts were compared using generalized mixed linear models. Statistical significance was adopted with p < 0.05.

The median age was 29.7 years. The average HGR of the participants, before interventions, was 2.35 mm per week (SD = 0.39 mm), consistent with the literature data. There was a significant increase (p = 0.02) both in HGR and in the photographic area covered by hair (p < 0.01) only in the group that used the combination of topical minoxidil and oral biotin (Table 1).

**Table 1:** Average hair growth rate evaluation by difference in hair length and photographic area covered by hair shafts’, before and after each intervention.

Intervention	Growth speed before intervention	Growth speed after intervention	p^c^
Evaluated by difference in hair length^a^			
Topical minoxidil 5%	2.63 (SD = 0.43)	2.57 (SD = 0.46)	0.46
Oral biotin 5 mg	2.50 (SD = 0.38)	2.47 (SD = 0.42)	0.53
Topical minoxidil 5% + oral biotin 5 mg	2.36 (SD = 0.36)	2.64 (SD = 0.32)	**0.02**
Evaluated by difference in area covered by hair shafts^b^			
Topical minoxidil 5%	2.33 (SD = 0.37)	2.30 (SD = 0.59)	0.55
Oral biotin 5 mg	2.22 (SD = 0.34)	2.68 (SD = 0.65)	0.28
Topical minoxidil 5% + oral biotin 5 mg	2.13 (SD = 0.73)	2.54 (SD = 0.4)	**<0.01**

a Growth speed in millimeters per week.

b Growth speed in increase of percent of area covered by hair shafts per day.

c Bold values were regarded significant.

Minoxidil increases hair thickness, prolongs the anagen phase, and promotes alternation from the telogen to anagen phase in patients with androgenetic alopecia.^2^, ^3^ A recent review suggests that its topical application activates molecular signaling pathways, such as beta-catenin, and stimulates prostaglandin E2 receptors, contributing to its positive effects on the hair cycle and growth.^2^, ^6^

Biotin is a cofactor in multiple metabolic pathways, with probable importance in nail growth and thickness.^3^ Notwithstanding, few studies have examined the efficacy of biotin in treating hair and nail disorders,^4^, ^7^ with most of the case series regarding its use in hair diseases. Randomized trials studying biotin supplementation in hair disorders are lacking, and its prescription still occurs without robust evidence.

In our study, the isolated use of both substances did not present a positive result in increasing growth velocity. It’s possible to deduce that biotin may act as a cofactor in the hair cycle, with a potential synergistic effect, modulating the effect of minoxidil or anticipating its effects on hair growth. Nevertheless, there is no previous study supporting this information.

We cite as limitations of the study the sample size, the influence of seasonality, and the sample group being formed exclusively by adult men, which limits the generalization of the results.

With this study, we were able to verify the synergistic effect of the use of topical minoxidil and oral biotin in healthy men in increasing HGR in the first 14 days of use. More studies are needed to analyze the benefit of long-term use of the combination, as well as to evaluate the isolated use of such medications in longer periods.

## Financial support

None declared.

## Authors’ contributions

Flávia de Oliveira Valentim: Conception and design of the study; data collection; article writing; critical review of the literature; final approval of the final version of the manuscript.

Anna Carolina Miola: Conception and design of the study; article writing; critical review of the literature; final approval of the final version of the manuscript.

Hélio Amante Miot: Conception and design of the study; statistical analysis; article writing or critical review of important intellectual content; final approval of the final version of the manuscript.

Juliano Vilaverde Schmitt: Conception and design of the study; data analysis and interpretation; statistical analysis; article writing or critical review of important intellectual content; effective participation in research guidance; final approval of the final version of the manuscript.

## Conflicts of interest

None declared.
